# Accurate Measurements and Simulations of the Evaporation and Trajectories of Individual Solution Droplets

**DOI:** 10.1021/acs.jpcb.2c08909

**Published:** 2023-04-07

**Authors:** Daniel A. Hardy, Joshua F. Robinson, Thomas G. Hilditch, Edward Neal, Pascal Lemaitre, Jim S. Walker, Jonathan P. Reid

**Affiliations:** † School of Chemistry, University of Bristol, Bristol BS8 1TS, United Kingdom; ‡ H. H. Wills Physics Laboratory, University of Bristol, Bristol BS8 1TL, United Kingdom; § Institut für Physik, Johannes Gutenberg-Universität Mainz, Staudingerweg 7-9, 55128 Mainz, Germany; ∥ Institut de Radioprotection et de Sûreté Nucléaire (IRSN), PSN-RES, SCA, LPMA, Gif sur Yvette 91192, France

## Abstract

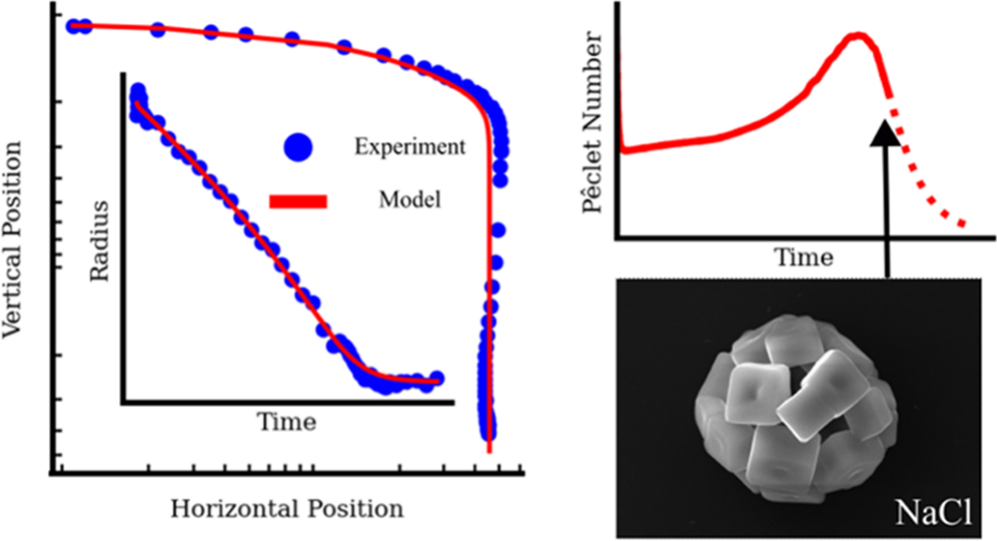

A refined numerical model for the evaporation and transport of droplets of binary solutions is introduced. Benchmarking is performed against other models found in the literature and experimental measurements of both electrodynamically trapped and freefalling droplets. The model presented represents the microphysical behavior of solutions droplets in the continuum and transition regimes, accounting for the unique hygroscopic behavior of different solutions, including the Fuchs–Sutugin and Cunningham slip correction factors, and accounting for the Kelvin effect. Simulations of pure water evaporation are experimentally validated for temperatures between 290 K and 298 K and between relative humidity values of approximately 0% and 85%. Measurements and simulations of the spatial trajectories and evaporative behavior of aqueous sodium chloride droplets are compared for relative humidity values between 0 and 40%. Simulations are shown to represent experimental data within experimental uncertainty in initial conditions. Calculations of a time-dependent Péclet number, including the temperature dependence of solute diffusion, are related to morphologies of sodium chloride particles dried at different rates. For sodium chloride solutions, dried particles are composed of collections of reproducibly shaped crystals, with higher evaporation rates resulting in higher numbers of crystals, which are smaller.

## 1 Introduction

The transportation of evaporating aerosol droplets plays a fundamental role in a wide range of industrial, health, and environmental applications.^1−4^ Such applications include industrial spray drying and surface coating, respiratory disease transmission, and atmospheric aerosol and cloud droplet transportation and dynamics.^5−9^ Across the wide variety of application areas, the underlying processes of heat and mass transfer between the droplet surface and surrounding gas phase, and the impact on droplet trajectory, are central. It is therefore valuable to provide an accurate and accessible approach to modeling the interplay between the physicochemical processes underlying droplet evaporation/condensation and transport.

Modeling evaporation/condensation in aerosol droplets is challenging because the heat and mass transfer between the droplet surface and surrounding gas phase are coupled.^8^ Indeed, including droplet transport into the model framework introduces further complexity because physical properties such as size and density, which have a significant impact on the aerodynamic properties, can change rapidly during evaporation. Despite these challenges, accurate models of single droplet behavior are an important step in advancing our understanding of the collective dynamics of an aerosol ensemble. The microphysical processes that determine droplet behavior may be explored more rapidly and systematically *in silico* than in physical measurements. Modeling allows the evolution of individual properties, which might not be directly measurable, to be predicted throughout the evaporation period. For example, the internal diffusion of solutes, which governs the level of surface enrichment during droplet evaporation (quantified using the Péclet number),^10^ is temperature- and concentration-dependent. Modeling enables the evolving droplet temperature and composition to be used to estimate a time-dependent Péclet number throughout an experiment. An additional benefit of a validated model is to guide the development and application of experimental approaches for measuring rates of droplet drying.^11^

Numerous model treatments of aerosol evaporation/condensation have been developed, using different approaches to treating the relevant microphysical processes. It is useful to separate these models depending on whether or not they account for the interplay between droplet motion, relative to the gas phase, in addition to heat and mass transfer. Models which consider evaporation of droplets within a stationary gas phase include a semianalytical approach by Kulmala.^12,13^ Although accurate for modeling evaporation in fairly humid environments, it becomes less accurate when the humidity drops below ∼80%RH. Under these dryer conditions, the evaporation rate increases and the associated evaporative cooling becomes significant, which is not fully accounted for. An advancement upon the Kulmala model was made by Kulmala, Vesala, and Heidenreich, to consider continuum regime condensation while including thermal diffusion and Stefan flow.^14,15^ A numerical description of heat and mass transport during droplet evaporation was developed by Su et al.^16,17^ The Su model accurately describes the temperature suppression of evaporating droplets, though it has not been widely implemented in other studies. The Kulmala and Su models do not account for inhomogeneity in droplet composition or temperature. This can be overcome with models incorporating internal concentration gradients that form during drying, though these typically do not simultaneously describe droplet transport.^18−21^ Recent work by Rezaei et al. has integrated surface enrichment effects and crust formation descriptions into droplet evaporation models,^22^ though this does not include fully coupled descriptions, depending instead upon analytical or semianalytical representations of heat and mass transfer.

A more complete model framework of droplet dynamics requires the coupling of heat and mass transport with droplet motion, since movement relative to the gas phase affects droplet evaporation and, consequently, changes the aerodynamic properties. Xie et al. developed a representative model for an evaporating droplet in motion, which continuously solved the coupled velocity, mass transfer, and heat transfer differential equations at discrete time steps.^23,24^ Lui et al. made improvements to the Xie model by including insoluble solids.^24^ Walker et al. adapted the Xie model to include thermodynamic treatments of solutions other than NaCl, such as deep lung fluid and saliva, to accurately describe the trajectories and settling times of respiratory droplets in cough jets.^25^

Routine use of a coupled evaporation and transport model, like those introduced above, is hampered by two factors. First, there is a lack of appropriate experimental single-particle data, stemming from a lack of suitable experiments with which to benchmark and validate the model. Second, there is currently no readily available computational package, implementing such a model, for common use. Single droplet evaporation models require careful validation against experimental measurements to ensure that representations are accurate, particularly when complex processes such as phase transformations (e.g., crystallization) occur during drying. Ideally, such measurements should be performed upon contact-free droplets to ensure surface effects are not present. Suitable techniques for investigating evaporation in stationary droplets include electrodynamic trapping instruments such as a comparative kinetics electrodynamic balance (CK-EDB).^11^ However, to fully benchmark model treatments that describe the interplay between droplet evaporation/condensation processes and transport requires accurate measurements on moving droplets. Such measurements are now available using the new falling droplet chain (FDC) technique which enables high time resolution measurements and detailed imaging of droplets undergoing crystallization.^26^

Here, we aim to test the most advanced single droplet coupled evaporation/condensation and transport model against the most accurate single droplet evaporation measurements available and confirm the correlation between the simulated and experimental data. Specifically, we introduce an updated coupled droplet evaporation/condensation and transport model, based on the models developed by Xie et al. and Walker et al., which evaluates the coupled behavior of droplet motion, temperature, and size change and provides time-resolved evaporation profiles and two-dimensional droplet trajectories.^23,25^ The model framework is validated with experimental data from the CK-EDB and FDC instruments. As with its predecessors, the model uses continuum regime descriptions of droplet–gas interactions. However, as an advancement, this model includes the Fuchs–Sutugin correction factor, Cunningham slip correction factor, and Kelvin effect. Such factors extend the range of droplet sizes at which the model is applicable from a lower limit of approximately 10 μm down to the order of 1 μm at standard temperature and pressure. As with the existing models, this approach also assumes droplet homogeneity in concentration and temperature.

In this article, we first present the underpinning theory and parameterizations of the model framework. We then compare the droplet evaporation calculations for stationary droplets against existing models and experimental CK-EDB data and show the capability for real-time assessment of RH within a CK-EDB chamber. We then assess the model performance against FDC measurements of individual droplets falling through a gas phase to validate the modeled calculations of droplets in motion, including a detailed sensitivity analysis. Additionally, we include a comparison between the expected and measured crystallization times, assuming prompt crystallization when a solute supersaturation threshold is reached. We then present a sensitivity analysis, assessing the sensitivity of simulations to the input parameterizations and variations in initial conditions, based on experimental uncertainties. Following validation, we then test the model to extract time-resolved Péclet numbers throughout the evaporation period and examine and compare the drying rates to observed final particle morphologies.

In this work, we have implemented the model using the Single Aerosol Drying Kinetics and Trajectories (SADKAT) software package.^27^ SADKAT is a bespoke, free-to-use, and open-source program, written using Python, which combines the model framework discussed in this paper with a convenient user interface. It allows complete droplet trajectories and evaporation profiles to be calculated within seconds of computational time on a typical personal computer. We anticipate that SADKAT will be readily adapted to new experiments to meet the needs of single-particle aerosol scientists, and able to act as a reference to verifying new numerical models. For simplicity, we label the simulations generated using the model as “SADKAT” throughout this article.

## 2 Theoretical Background

The model is capable of representing evaporation and condensation processes for droplets of binary solutions, consisting of one volatile component and an involatile component (or a mixture of involatile components that can be represented as a single component), typically an inorganic salt. The influence of dissolved solutes on the vapor pressure of the solvent, known as the solute effect, is accounted for by relating the composition of the droplet, in terms of standard molality, to the solvent activity with an activity coefficient, *γ*. The model also includes the capability to account for nonideal hygroscopicity as implemented by Walker et al.^25^

The model treats the gas surrounding a droplet as a continuous fluid. This assumption can break down if the size of droplets is small compared to the mean free path, *λ*, of gas phase molecules. The model presented by Xie et al. was implemented such that simulations were terminated if droplets evaporated to 0.3 μm in radius.^23^ To assess if it is appropriate to consider the gas phase as continuous, the Knudsen number, *Kn*, is used, defined in Equation 1. The Knudsen number is the ratio of the mean free path of the surrounding gas to the radius of a particle, *r*. Particles with *Kn* ≪ 1 are in the continuum regime, and particles with *Kn* ≫ 1 are considered in the free molecular regime, where the gas is not considered as a continuous fluid. Particles with *Kn* ≈ 1 are considered to be in the transition regime, where the continuum approach may be used with the inclusion of correction factors.^28^ Our model has been developed for application to droplets in the continuum regime, but correction factors, such as the Fuchs–Sutugin correction factor, have been included to extend the range of validity to smaller droplet sizes.
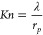
1

The framework assumes droplets are both thermally and compositionally homogeneous and the effects of surface enrichment are not accounted for in any way, with no description of solidification behavior of any sort, such as crystallization or crust formation.

Within the bounds discussed, the state of a droplet may be described using the equations presented by Xie et al. which are also shown in the Supporting Information in eqs S1–S4.^23^ When considered together, these equations describe the evolution of droplet mass, *m*_p_, droplet temperature, *T*_p_, droplet velocity, *V*_p_, and droplet position, *x_p_*, with respect to time, *t*, respectively. This system of ordinary differential equations are numerically integrated over time, using dynamically selected time steps.

### 2.1 Accurate Parameterization of Physicochemical Quantities

To describe solution droplet transport and evaporation processes accurately, parameterizations of the physicochemical properties of the system are required. These are considered in three categories: environmental, solvent, and solution properties.

Interactions with the gas phase determine the processes that occur at the droplet surface. Similarly, the transport of a droplet depends upon the nature of the gas it is surrounded by. As such, gas phase conditions need to be described in detail, with temperature-dependent parameterization where necessary. The surrounding gas in the experiments presented in this work is air, though any other gas phase may also be used, by including the appropriate parameterizations. A representative molar mass of air of 28.9647 g/mol and a standard atmospheric pressure of 101325 Pa are used. The density of air is parameterized with respect to temperature using the parameterization by Lemmon et al. included in the Chemicals python library.^29,30^ The dynamic viscosity and thermal conductivity of air are parameterized as recommended by Lemmon and Jacobsen.^31^ The specific heat capacity of air is taken as 1006 J/kg/K.

**Figure 1:**
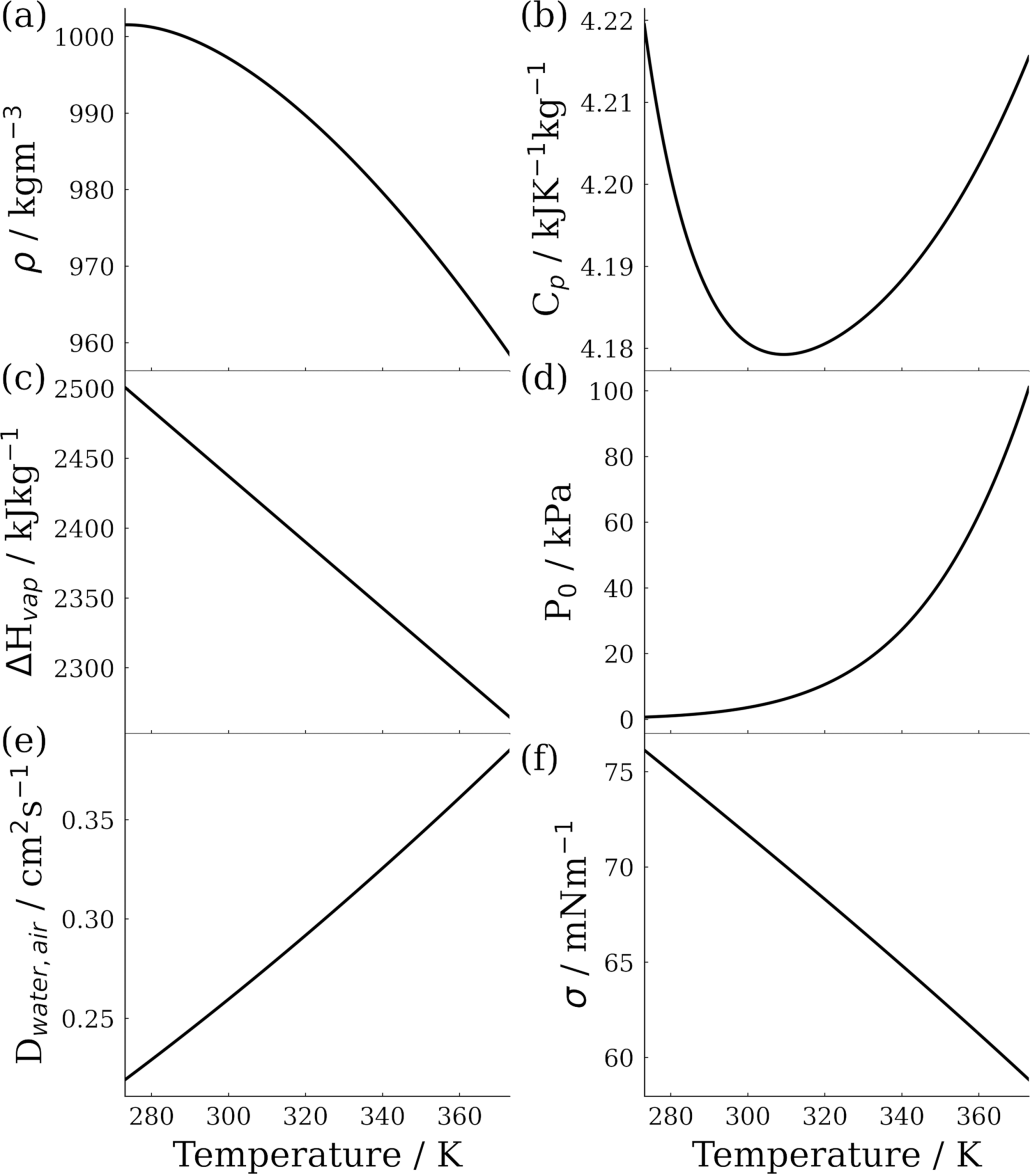
Parameterizations of bulk properties of water with respect to temperature. (a) Density, (b) specific heat capacity, (c) enthalpy of vaporization, (d) vapor pressure, (e) binary diffusion coefficient of water vapor in air, and (f) surface tension.

The physicochemical properties of the liquid and gaseous states of the evaporating volatile solvents must be accounted for to describe gas phase diffusion limited evaporation, where internal transport mechanisms do not limit solvent molecules from reaching the droplet surface. In this work, the solvent used is water, however, the model is capable of using any other solvent, given the appropriate parameterizations. The equilibrium vapor pressure of water is parameterized as a function of temperature using functional form and coefficients suggested by Buck.^32,33^ The density of liquid water is defined using the parameterization provided by Wagner et al., a fractional power series, scaled by a reference density.^34^ The specific heat capacity of water is described using an implementation of the IAPWS-95 standard and the approach presented by Sippola et al.^30,34−36^ The latent heat of vaporization of water is parameterized with respect to temperature linearly, using the same coefficients as Su *et al*.^17^ The binary diffusion coefficient for water vapor is parameterized using the same functional form as Xie *et al.*, presented in Equation 2.^23^*D*_ref_, *T*_ref_, and ξ are determined empirically, and values of 0.2190 × 10^–4^ m^2^ s^–1^, 273.15 K, and 1.81 are used, respectively. The surface tension of water is described using the DIPPR Equation 106 and coefficients recommended by the Dortmund Data Bank.^37−40^
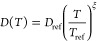
2The parameterizations of water’s properties are shown in Figure 1.

**Figure 2:**
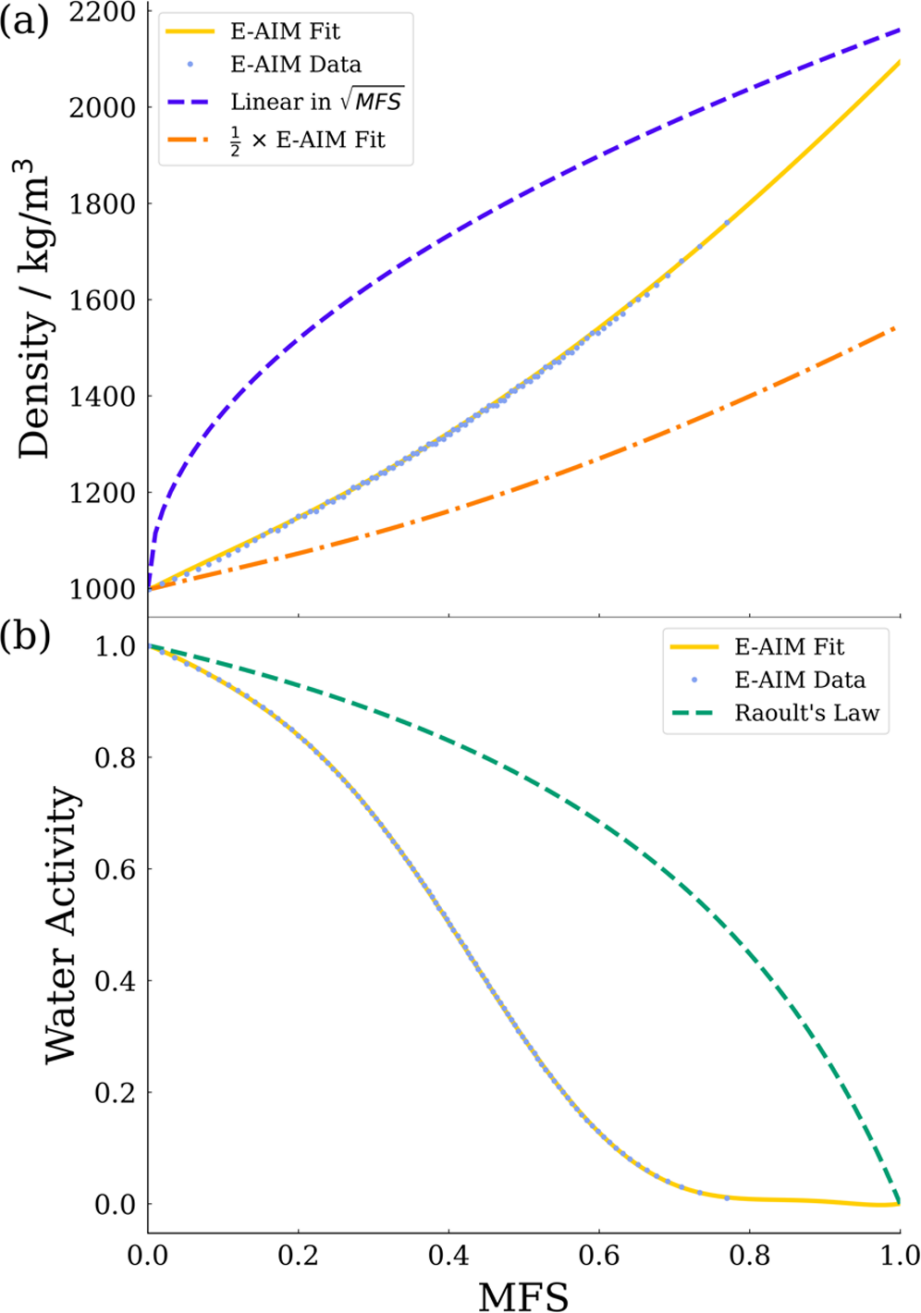
(a) Density parameterizations of aqueous sodium chloride solutions with respect to MFS. Density data from E-AIM are used to produce a polynomial fit. A simplified fit, linear in MFS^1/2^ between the density of pure water (MFS = 0) and the solid density of NaCl (MFS = 1), is included. Additionally, a reduced density system is represented using a parameterization of a half-times the E-AIM fit.

The properties of a solution can be dramatically different from those of the pure solvent. As droplets evaporate, the concentration of involatile components increases. To account for this, parameterizations of the properties of a solution over a wide range of concentrations must be considered. In this work, the solution presented is aqueous NaCl, a system which has already been well parameterized in the literature. The solution density is parameterized using a polynomial with respect to the square root of solute mass fraction as described by Clegg and Wexler.^41,42^ This polynomial is fit to data from the Extended Aerosol Inorganics Model (E-AIM).^43,44^ To test the sensitivity of simulations to droplet density alternative parameterizations are also used: a linear parameterization in the square root of mass fraction solute (MFS) domain, a simple representation, and a polynomial with a gradient half that of the fit to E-AIM, to approximate droplets that form hollow particles with low densities. Solvent activity is parameterized in the MFS space using a constrained polynomial, forced to pass through (0,1) and (1,0), corresponding to the pure component solvent and a theoretical state of pure component solute. This polynomial is fitted to values extracted from experimental data obtained, for example, from CK-EDB measurements.^45^ In the case of NaCl these data may be obtained from the E-AIM model.^43^ Xie et al. assumed that solvent activity obeyed Raoult’s law and is linear in mole fraction; Walker et al. implemented the nonideal representation of activity.^23,25^ Raoult’s law is also included for comparative simulations here. Solution properties, such as solvent activity, are parameterized with respect to MFS. This is convenient for comparison with experimental work and often performed using MFS measurements. The parameterizations of solution density and solvent activity for aqueous sodium chloride solution used are shown in Figure 2. It is seen that both the linear parameterization of density with respect to MFS^1/2^ and Raoult’s law present as nonlinear in the MFS parameter space.

## 3 Experimental Methods

The model described above is validated by comparison with two different experimental approaches: the CK-EDB and the FDC. These are cutting-edge techniques that interrogate stationary and moving droplets, respectively. Each technique has associated advantages; the CK-EDB is able to collect very precise size data through light scattering measurements but is unable to resolve the aerodynamic size of particles, the FDC collects lower precision size data, but enables direct imaging of particles throughout drying, the collection of a sample of dry particles, and measurement of aerodynamic size. The combined usage of two techniques enables thorough benchmarking to be performed and a high level of physical insight to be gained.

### 3.1 Comparative Kinetics Electrodynamic Balance

The CK-EDB allows robust and accurate measurements of the evaporation dynamics of individual stationary droplets. The technique has been introduced in more detail previously, so here we just outline the main components.^11^ The instrument consists of parallel concentric cylindrical electrodes within a chamber, through which a gas of controlled speed, temperature, and RH is flowed. A piezoceramic droplet-on-demand (DoD) dispenser generates highly reproducible, single, micron-sized droplets from an inductively charged solution, which are then electrostatically confined in an oscillating electric field. The time taken between droplet generation and confinement is ∼0.1 s.^46^

In this work, the evaporation dynamics of individual droplets of pure water levitated in a CK-EDB is measured. Water droplets are trapped and evaporation is measured at temperatures between 290 and 298 K and 0% RH and 85% RH. The droplet sizes are measured from laser light scattering using the procedures described in previous work.^11,19^ An initial radius, *R*_0_, is calculated for each droplet by a linear back-extrapolation in *R*^2^ to *t* = 0 from the earliest measurement point. Repeat measurements are made and the mean initial size is used as the initial value for simulation. The standard deviation in the mean is taken as the uncertainty and this is always less than 0.1 μm.

The RH within the CK-EDB chamber is held at a constant value for each experiment and is inferred using a probe droplet. In humid conditions (>80%RH), the evaporation rate of water droplet can be compared to the Kulmala model to resolve the RH with a high degree of accuracy. However, large uncertainties are introduced under dryer conditions (explored in more detail below), as evaporative cooling is not well accounted for by the Kulmala model. Below 80%RH, the equilibrium size of a NaCl droplet can be used to calculate the RH. However, the use of an inorganic salt is limited to RH values above the efflorescence RH (∼45% RH for NaCl). Below this value, the droplet crystallizes and retrieval of an accurate size is not possible. Although other inorganic salts, such as LiCl (efflorescence RH = 11.3% RH), can be used to probe dryer conditions, there are associated disadvantages as not all salts are well characterized by thermodynamic models.^47^ As with initial radius, the mean RH from repeat experiments is used to set the RH for the model simulations, with the standard deviation taken as the associated uncertainty. To acquire data for comparison with the model at a very low RH, a gas flow of dry nitrogen gas (0% RH) is used. Under these conditions, it is not possible to measure the RH using a probe droplet in the existing systems. As the EDB is not a fully closed system, it is not possible to ensure that the RH experienced by the droplets is 0%. Electronic RH probes with an associated error of ± 5% at ∼ 0% RH have been used to validate the low RH in the EDB chamber and so the RH is taken to be between 0 and 5%. In all experiments, the temperature is measured using a thermocouple probe with ± 1 K uncertainty.

### 3.2 Falling Droplet Column

**Figure 3:**
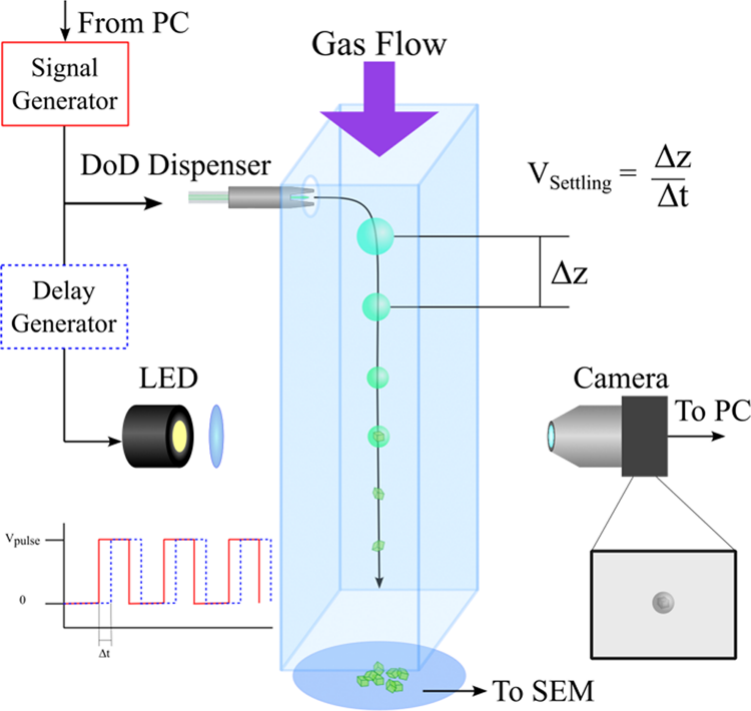
Simplified representation of the FDC showing the key components used in the generation and imaging of droplets and the collection of dry particles.

The FDC offers a unique method for measuring the coupled evaporation and transport dynamics of falling droplets, convenient for comparison against our model simulations. The FDC has been introduced in detail previously, so here we just outline the main components.^26^ Individual droplets are generated at a regular frequency (10s of Hz) using a DoD dispenser. The droplets propagate down through a vertical column in line with a gas flow of controlled flow rate and RH, as indicated in Figure 3. Droplets are imaged using stroboscopic imaging, enabling a high time resolution and sampling of multiple droplets at a given time-point after generation. Geometric diameter is measured directly from calibrated images of droplets, using a calibration factor with units of meters per pixel. FDC measurements enable the trajectories of droplets to be analyzed concurrently with the droplet size evolution. In this work, measurements of the evaporation of falling droplets of aqueous NaCl, with an initial concentration of 0.05 MFS and a radius of ∼20 μm, are made over a range of RH values for comparison against the model. The RH and temperature are measured using capacitance and thermocouple probes, respectively.

The initial conditions for running the simulations (RH, T, initial size, initial droplet velocity vectors, and gas flow dynamics) are extracted from the experimental FDC data. To evaluate the initial droplet horizontal and vertical velocity, consecutive measurements of droplet position with a known temporal separation (typically 1 × 10^–5^ s for such measurements) are made. Droplets generated from DoD dispensers oscillate for a short period immediately after generation, making the definition of droplet position and size difficult. A small uncertainty in droplet position corresponds to a large uncertainty in velocity when using small time steps. To reduce uncertainty and achieve a representative set of initial conditions a mean of the five earliest measured values of droplet velocity and size is used as initial values for simulations. All of the initial conditions used in this work (shown in Table 2) are taken within 0.5 ms of droplet generation. The uncertainty is taken as the standard deviation in the mean. The FDC technique allows the collection of dried particles. Particles deposit upon a glass collection slide which may be removed from the FDC for further analysis, such as SEM.

## 4 Results and Discussion

### 4 1 Intercomparison with Existing Models

To assess the model performance, we initially compare simulated steady-state evaporation rates of single pure water droplets, over a range of ambient RHs, against existing models. The evaporation rate, κ, is defined in eq 3 as the rate of change of the square of droplet diameter, *2r*_p_, over time, *t*. An alternative expression in terms of droplet radius is also shown.^48^
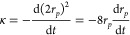
3

Although evaporating droplets exhibit unsteady evaporation immediately after generation, this phenomenon is typically only observed in simulations and not in the measurements used in this work, as it requires overcoming the challenge of measuring the evaporation rate at very early time points. During steady-state evaporation, κ is constant and may be evaluated from experimental data as the mean value of d(2*r*_p_)^2^/d*t*.^49^ To capture a representative value of steady-state evaporation of droplets in the continuum regime from the modeled simulations, a mean evaporation rate is calculated after excluding the initial and final 10% of droplet lifetime.

**Figure 4:**
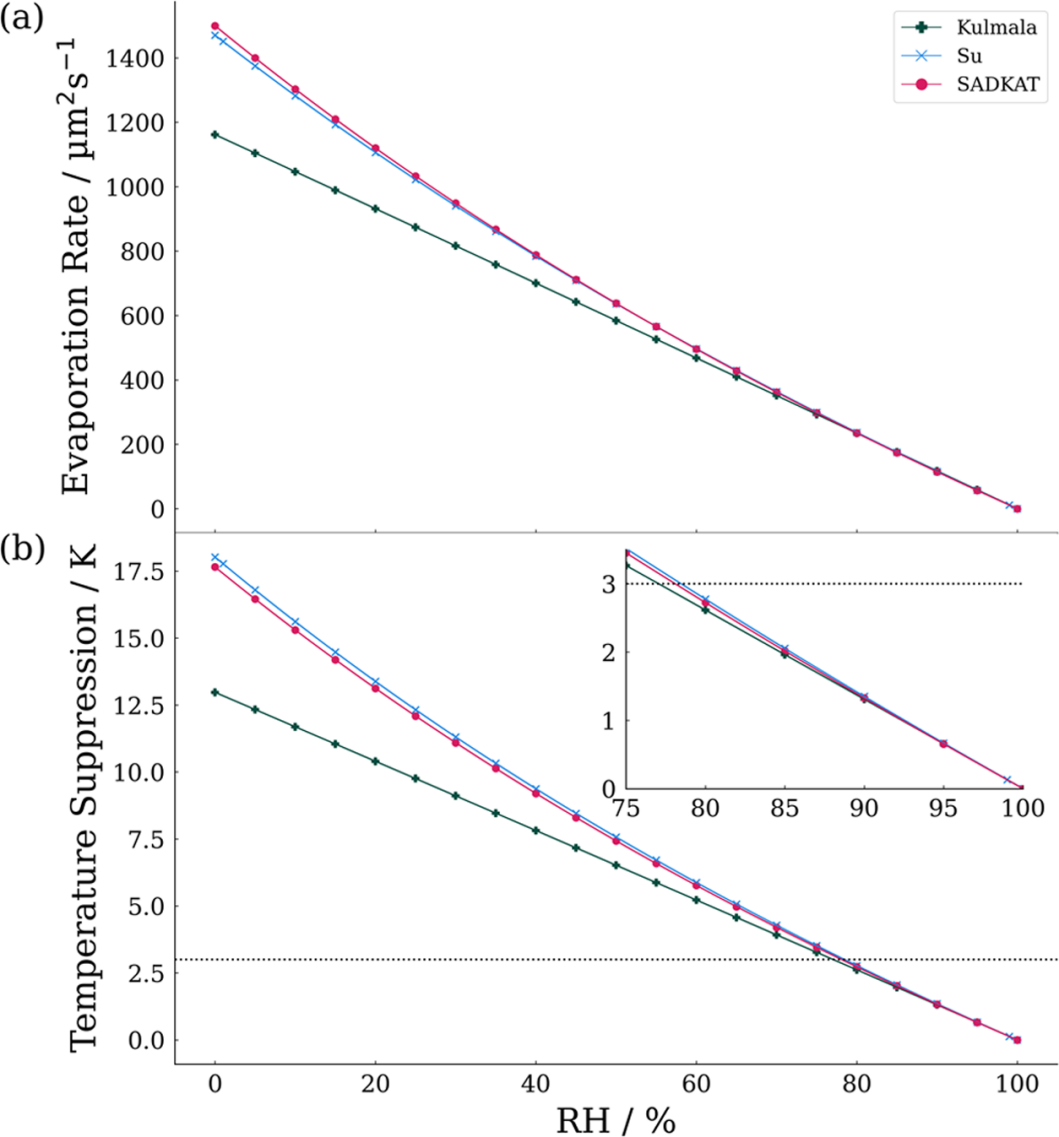
(a) Comparison of calculated evaporation rate according to eq 3 using Kulmala model, Su model, and SADKAT. (b) Calculated temperature suppression of droplets during steady-state evaporation using Kulmala model, Su model, and SADKAT. A black dotted line is included at 3 K for reference.

Figure 4a is a comparison of the steady-state evaporation rate of pure water droplets with an initial radius of 25 μm calculated using our model and the Kulmala and Su models, at 298.15 K over the RH range from 0 to 100%. The three models calculate an evaporation rate of 0 μm^2^ s^–1^ at 100% RH and diverge at lower RH values. In particular, the Kulmala model clearly deviates from the other models below 80% RH. This is a consequence of approximations made in the Kulmala model when calculating the droplet temperature. Indeed, in the literature, the description of temperature suppression in the Kulmala model is only considered accurate when the difference between the gas phase temperature and droplet temperature is less than 3 K.^17^ Our model closely aligns with the experimentally validated Su model across the RH range. Figure 4b presents the calculated temperature suppression (*T*_gas_ – *T*_droplet_) of droplets during steady-state evaporation by the three models. Measurement of the temperature of evaporating water droplets has been achieved using Raman scattering and laser-induced fluorescence, but such measurements are not available for many systems and are technically challenging.^50,51^ Consequently, simulations provide a convenient route to the evaluation of droplet temperatures during evaporation.

### 4.1 Intercomparison with Experimental Data

We now turn our attention to compare the model performance against high-precision single-droplet experimental data, first for stationary droplets (using the CK-EDB), and then for falling droplets (using the FDC).

#### 4.2.1 Evaporation of Stationary Droplets of Pure Water

**Figure 5:**
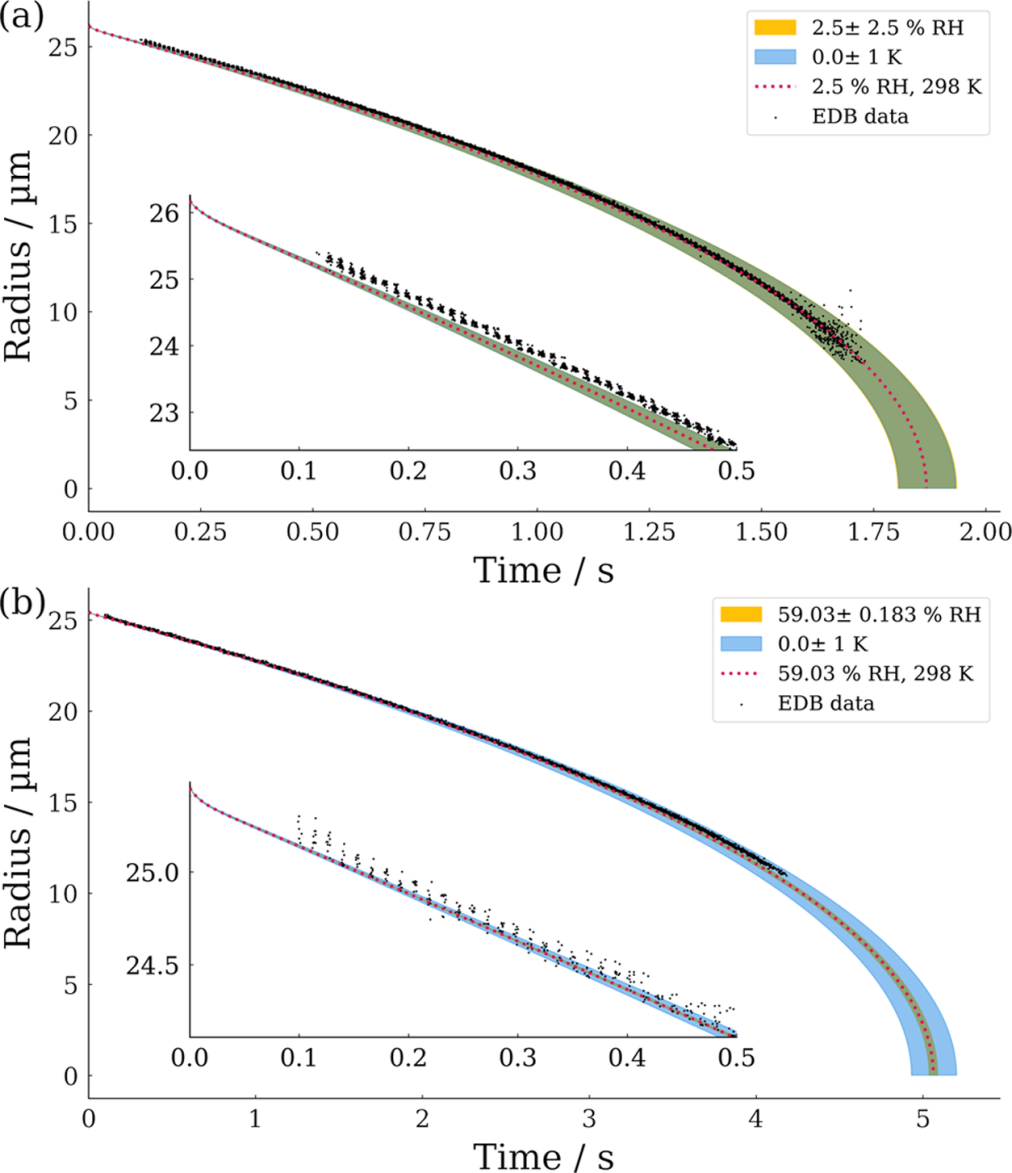
Comparison of CK-EDB measurements (black points) and SADKAT simulations (red dotted line) for the evaporation of water droplets at (a) 298 ± 1 K and 2.5 ± 2.5% RH and (b) 298 ± 1 K and 59 ± 0.2% RH. Yellow and blue envelopes correspond to simulations based on the uncertainty in initial conditions of RH and temperature, respectively.

Figure 5 is a comparison of the time dependence of the droplet radius between CK-EDB measurements and model simulations at 298 K at approximately 60% RH and 0% RH. Given the uncertainty in the EDB RH at ∼0%, simulations were performed using an RH of 2.5% with an uncertainty of ±2.5%. In both cases, the experimental data agree well with the simulations. This close alignment suggests that the true experimental conditions are well within the experimental uncertainties described above. In Figure 5a, the linearly extrapolated initial radius can be seen to be a source of error as it does not account for the period of unsteady evaporation. Experimental measurements are terminated before complete evaporation as droplets become unstable and are typically lost from the trap when they reach a sufficiently small size. For rapidly evaporating droplets, the final measurement points exhibit noise, potentially due to involatile impurities introducing uncertainties during the size retrieval, but this does not lead to significant deviations from the simulated trend.

**Figure 6:**
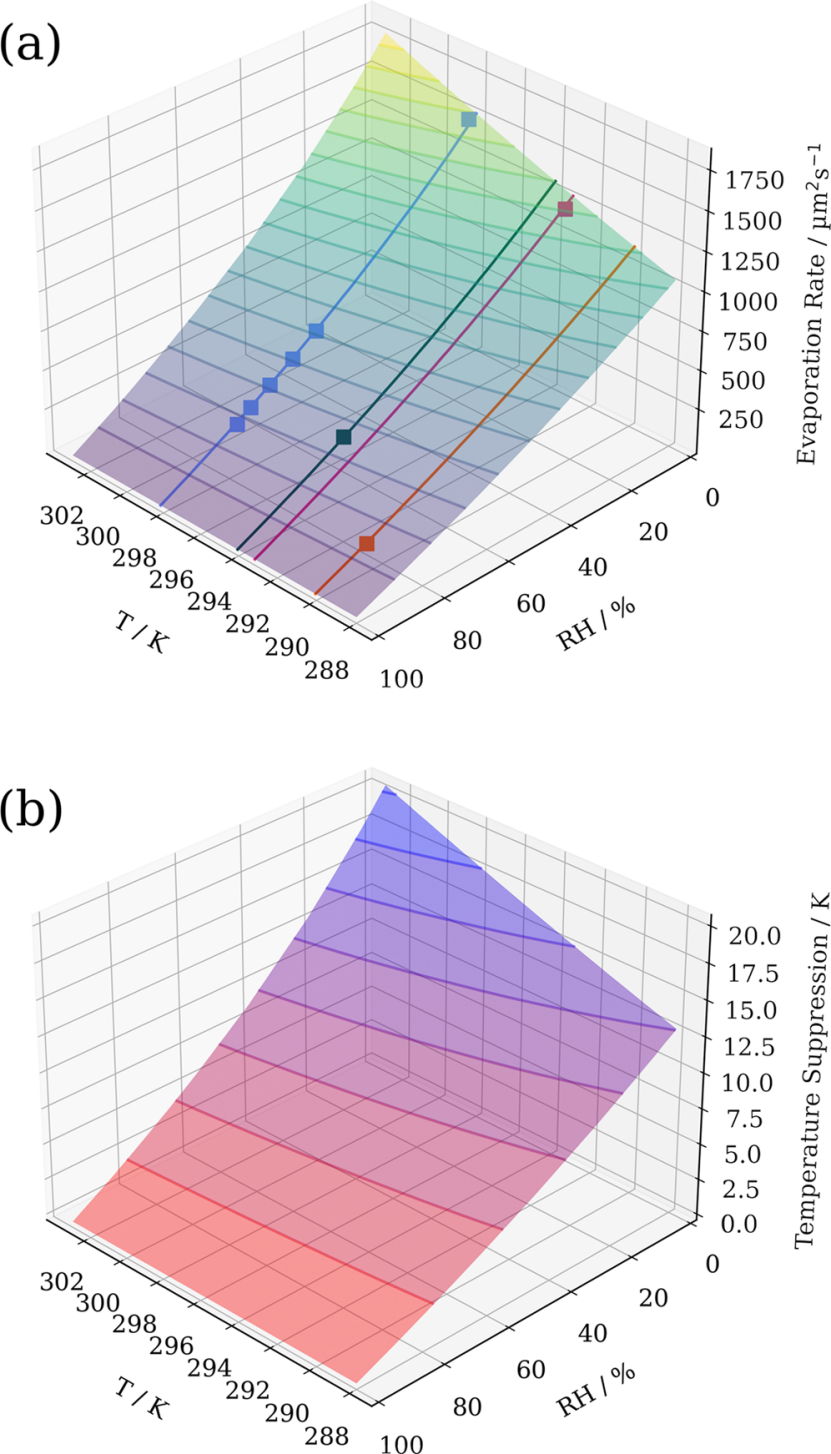
Properties of evaporating droplets calculated using SADKAT over the RH range 0–100% and temperature range 288–303 K. (a) Evaporation rate and experimentally measured values using a CK-EDB for comparison. Contours are marked every 100 μm^2^ s^–1^ and experimentally measured evaporation rates are marked with square points. Experimental data points are colored according to the temperature of the measurement, and the line along the surface of simulated results is marked with a solid line of the same color. (b) Temperature suppression calculated as environmental temperature minus droplet temperature, contours are marked every 2.5 K.

To assess the agreement between experiments and simulations more widely, experimental evaporation rates are compared to simulations across both the temperature and RH parameter space. This is shown in Figure 6a and is calculated in the same way as previously described. A comparison of measured and simulated evaporation rates is reported in Table 1. The calculated droplet temperature suppression throughout evaporation is presented in Figure 6b.

**Table 1:** Summary of CK-EDB Experiments^a^

*T*/K	RH/%	*R*_0_/μm	κ_CK-EDB_/μm^2^s^–1^	κ_SADKAT_/μm^2^ s^–1^
290 ± 1	84.6 ± 0.1	25.34 ± 0.03	35 ± 1	36 ± 1
293 ± 1	0–5	25.29 ± 0.05	301 ± 3	313 ± 12
294 ± 1	67.5 ± 0.2	25.38 ± 0.02	89.2 ± 0.5	89 ± 3
298 ± 1	0 – 5	26.18 ± 0.03	364 ± 13	377 ± 14
298 ± 1	51.7 ± 0.2	25.50 ± 0.03	152 ± 1	155 ± 4
298 ± 1	59.0 ± 0.1	25.45 ± 0.02	126 ± 1	128 ± 3
298 ± 1	66.2 ± 0.2	25.28 ± 0.04	103 ± 1	103 ± 3
298 ± 1	72.2 ± 0.2	25.24 ± 0.08	81 ± 2	83 ± 2
298 ± 1	76.4 ± 0.7	25.39 ± 0.01	65 ± 2	70 ± 2

a Temperature Error is quoted as the uncertainty associated with a thermocouple probe. RH error is taken as the standard deviation in the mean value calculated using a NaCl probe droplet, except where RH < 40%, Here, RH is expected to be less than 5%, but cannot be measured using a probe droplet. *R*_0_ is the mean initial size of droplet and the error is the associated standard deviation. κ_Ck_-Edb is the mean of the value calculated for each droplet in a given set of conditions with the uncertainty taken as the standard deviation. The uncertainty in κ_SADKAT_ is calculated from simulations with initial conditions based on experimental uncertainties.

The simulated evaporation rates of water droplets may be used as a route to characterize the RH in experimental systems. The measured evaporation rate of droplets may be compared to the surface in Figure 6a and, with a known temperature, the RH may be inferred. This is discussed in more detail in the Supporting Information.

#### 4.2.2 Evaporation of Freefalling Aqueous NaCl Droplets

We now compare the model against experimental data derived from falling droplets acquired using the FDC. Seven experiments were performed in the FDC and are compared to simulations. The parameters used as initial inputs for the simulations (RH and temperature, initial horizontal and vertical velocities, initial radius, and speed of the gas flow in the FDC) are shown in Table 2, alongside the associated uncertainties. Simulations are terminated at the time corresponding to the final measurement from the FDC.

**Table 2:** Experimental Conditions and Initial Conditions from Experiments Performed in the FDC and Used as Inputs for Model Simulations^a^

experimental RH/%	*T*/K	*V_x_*/ms^–1^	*V_z_*/ms^–1^	*R*_0_/μm	gas flow/ms^–1^
0 ± 5	294 ± 1	1.4 ± 0.2	0.0 ± 0.3	19.6 ± 0.2	0.007 ± 0.001
5 ± 5	294 ± 1	1.5 ± 0.2	0.0 ± 0.1	19.3 ± 0.2	0.008 ± 0.001
10 ± 5	294 ± 1	1.2 ± 0.2	0.24 ± 0.09	18.7 ± 0.4	0.007 ± 0.001
20 ± 5	294 ± 1	2.4 ± 0.5	0.15 ± 0.07	17.9 ± 0.2	0.006 ± 0.001
30 ± 5	294 ± 1	1.5 ± 0.3	0.0 ± 0.1	19.4 ± 0.3	0.007 ± 0.001
35 ± 5	294 ± 1	0.9 ± 0.2	0.0 ± 0.1	21.9 ± 0.6	0.005 ± 0.001
40 ± 5	294 ± 1	2.53 ± 0.05	0.3 ± 0.6	18.0 ± 0.2	0.005 ± 0.001

a RH and *T* are Quoted with Instrumental Uncertainties, Other Values are Mean Values of Initial Measurements with Associated Standard Deviations.

**Figure 7:**
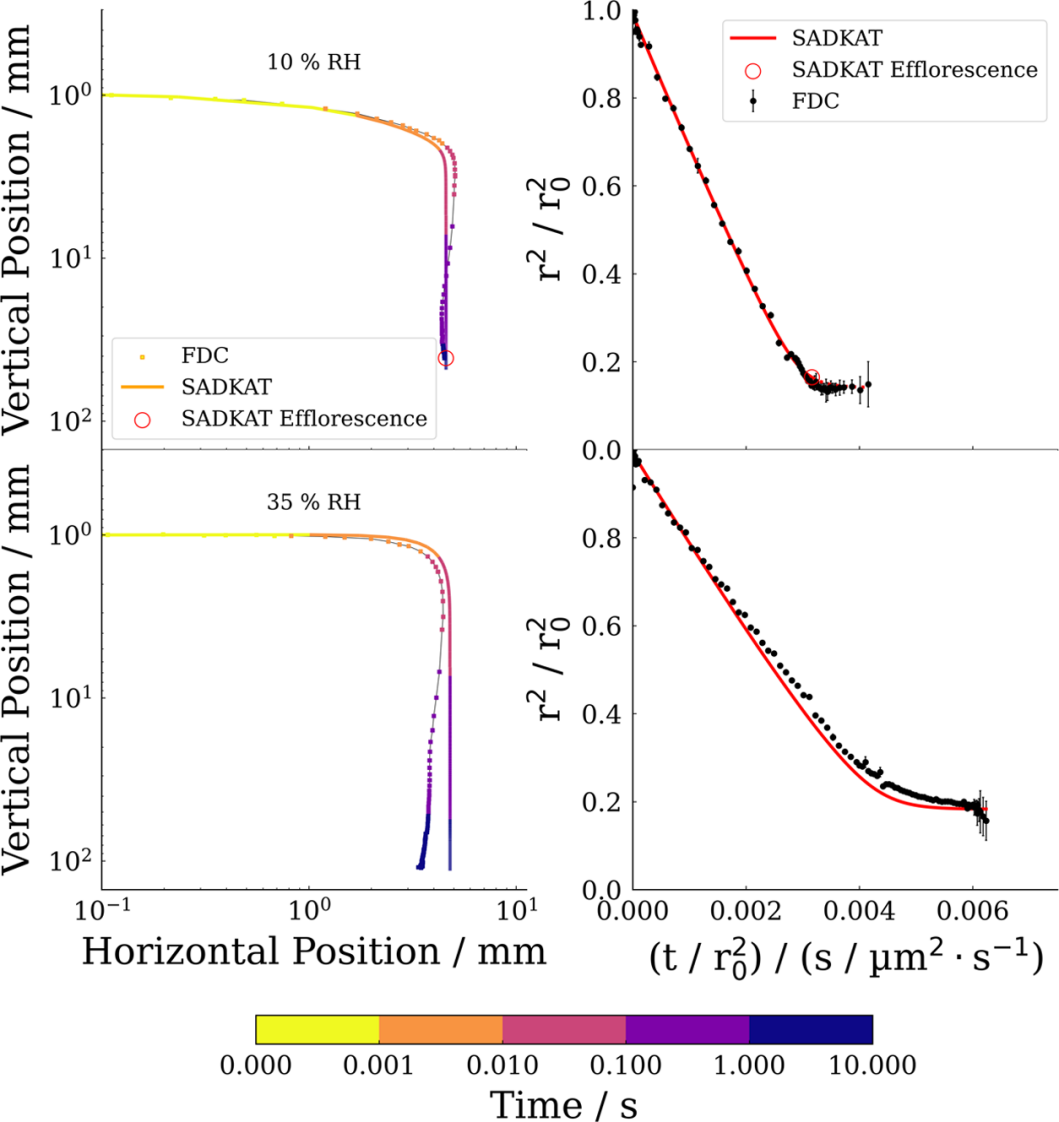
Comparative spatial trajectories (left) and evaporative profiles (right) of NaCl solution droplets (0.05 MFS) measured with the FDC and simulated using SADKAT at 294 K. The RH of each experiment is marked in the left-hand-side panel. The time of data points within the spatial trajectories is color mapped to a logarithmic scale with transitions at 1, 10, 100, and 1 s: *t* < 0.001 s (yellow), 0.001 s < *t* < 0.01 s (orange), 0.01 s < *t* < 0.1 s (pink), 0.1 s < *t* < 1 s (violet), 1 s < *t* (indigo).

Example experimental and simulated evaporation profiles and trajectories for NaCl droplets, evaporating 10 and 35% RH are compared in Figure 7. The full range of results for experimental RH values ranging from 0% RH to 40% RH are shown in the Supporting Information, Figure S1. The size changes are normalized with respect to the initial radius squared to enable comparison of the evaporation rates between different experiments. Droplet images from very early in the droplet lifetime can exhibit blurring, due to the high droplet velocity. This can sometimes artificially increase the measured initial geometric size (subsequently used in the simulations), resulting in some experiments displaying a systematic offset in radius between experiments and simulations. There are two possible reasons why the measured trajectories of the falling droplets sometimes deviate slightly from a truly vertical freefall (as can be seen in Figure 7). First, a slight misalignment between the axis of the imaging system and the column would systematically affect the measured trajectory. Second, the orifice through which the droplets are dispensed into the column perturbs the gas flow through the column. This is responsible for the apparent curved trajectories of experimentally measured trajectories. Despite this, from Figure 7, we can see that the measured trajectories and evaporation profiles are well captured by the model.

The crystallization time, taken as the time required for the water activity within the droplet to reach a given efflorescence threshold, can also be calculated from the model. In this work, the efflorescence threshold used is *a*_w_ = 0.24, corresponding to the activity of water in a NaCl solution at 2.04 time the saturation limit (2.04 × 0.26 MFS), when spontaneous nucleation in surface enriched drying droplets was reported by Gregson et al.^19,43^ Despite the model not including a representation of surface enrichment, the predicted crystallization time correlate well with experiments for experiments at a low RH (0–20%) and rapid evaporation rates. Above 20% RH, the observed and simulated crystallization times are seen to diverge, as shown in Table 3.

**Table 3:** Observed and Predicted Crystallization Time Measured in the FDC and Simulated Using SADKAT^a^

experiment RH/%	*t*_crys, obs_/s (±0.05 s)	*t*_crys, SADKAT_/s	(*t*_crys, obs_/*r*_0_^2^)/(s/μm^2^)	(*t*_crys, SADKAT_/*r*_0_^2^)/(s/μm^2^)
0	0.95	1.02	0.0025 ± 0.0001	0.0027
5	1.00	1.08	0.0027 ± 0.0001	0.0029
10	1.10	1.11	0.0031 ± 0.0001	0.0032
20	1.25	1.31	0.0039 ± 0.0002	0.0041
30	1.60	2.46	0.0043 ± 0.0001	0.0066
35	2.90	3.42	0.0060 ± 0.0001	0.0071
40		2.65		0.0082

a Absolute times are presented, and times normalized with respect to the square of the initial radius, which removes size dependence.

Gregson et al. demonstrated that rapidly evaporating NaCl droplets have concentrations that increase quickly and surpass the bulk saturation limit, with surface concentrations becoming significantly larger than the center only just prior to crystallization. This fast increase in surface saturation only immediately prior to crystallization may account for the similarity between the simulated and measured data, despite the model not accounting for surface enrichment effects.

### 4.3 Sensitivity Analysis

As discussed above, various experimentally derived input parameters are required to generate model simulations for comparison against FDC-acquired data. These include the environmental conditions, parameters relating to the droplet generation dynamics, and the initial bulk properties of the solution. Next, we perform a sensitivity analysis to assess the sensitivity of the model to each of these input parameters. Specifically, from a single FDC-acquired evaporation experiment, we generate multiple simulations, with the simulations using input parameters taken from the experiment. However, for each simulation, we artificially set the value for different input parameters to the extreme of the anticipated uncertainty and we then assess how this affects the comparison between the simulation and measurement. The FDC measurement used for this sensitivity analysis is the NaCl_(aq)_ droplet evaporation at 10%RH and the initial conditions, and the associated uncertainties, are shown in Table 2.

**Figure 8:**
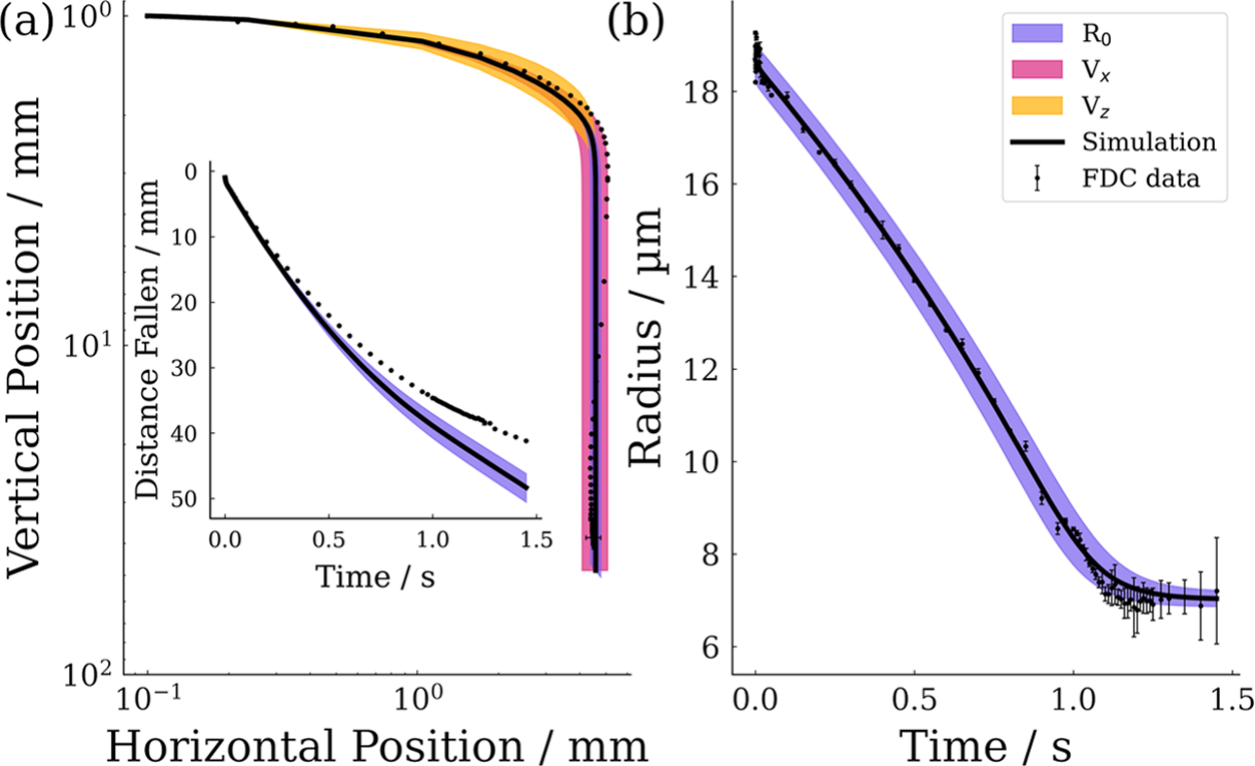
Sensitivity analysis of SADKAT simulations displaying the impact of the uncertainty in the initial conditions, initial radius, *R*_0_, horizontal velocity, *V*_*x*_, and vertical velocity, *V*_*z*_, upon results with FDC data shown for comparison. (a) Spatial trajectory of droplets with inset of distance fallen over time. (b) Evaporation profile of droplets.

Figure 8 highlights the sensitivity of the model to uncertainties in the droplet generation properties. As can be seen, the initial radius is the dominant source of uncertainty in both radial evolution and settling velocity. In Figure 8b, the uncertainty in the radial evolution due to the initial velocities, *V*_*x*_ and *V*_*z*_, is within the thickness of the line. The initial horizontal velocity is the key factor in determining the horizontal turning distance, while the initial vertical velocity has a significant positional impact only during the very early stage of droplet lifetime, while the particle is still relaxing to a terminal settling velocity.

**Figure 9:**
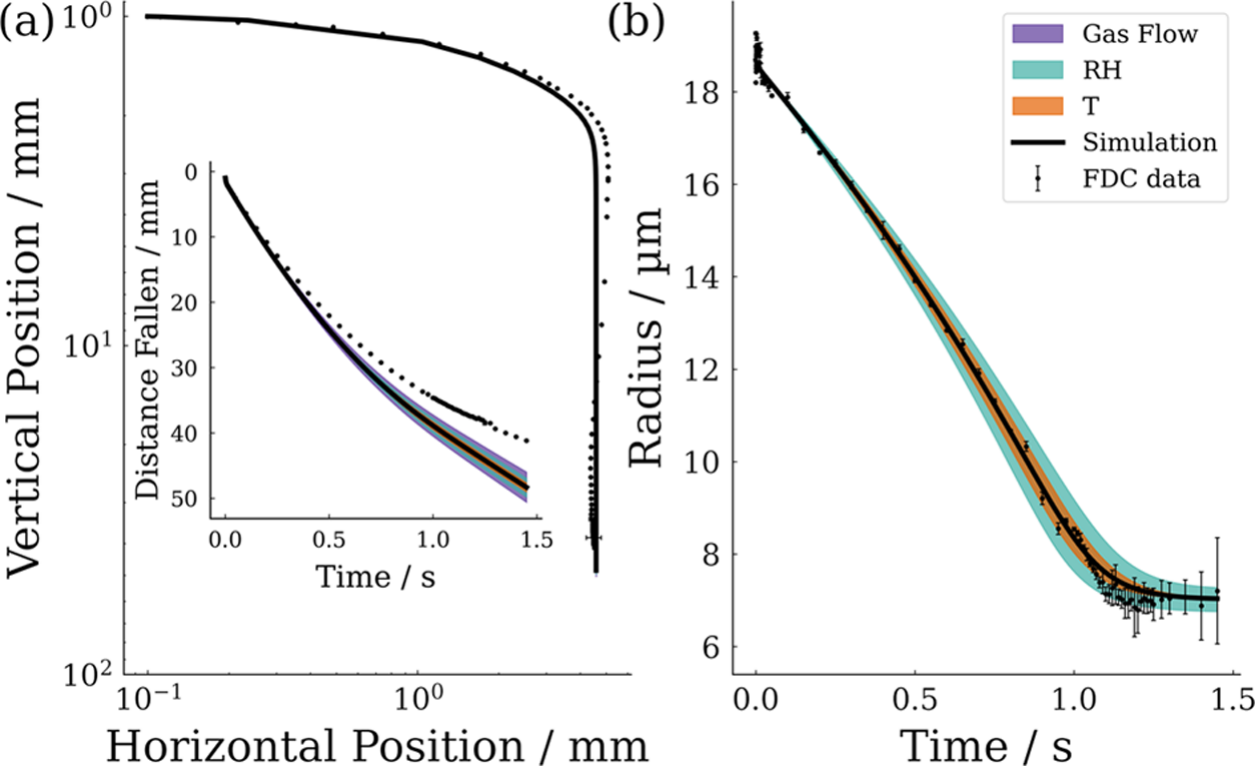
Sensitivity analysis of SADKAT simulations displaying the impact of the uncertainty in the initial conditions, gas flow, RH, and environmental temperature upon results with FDC data shown for comparison. (a) Spatial trajectory of droplets with inset of distance fallen over time. (b) Evaporation profile of droplets.

Figure 9 presents the sensitivity of the model to uncertainties in the environmental conditions. The speed of the gas flow is the dominant environmental source of uncertainty in the droplet trajectory, while the RH dominates uncertainties in droplet size. This is to be expected from eq S1 as the rate of change in mass depends directly upon the environmental vapor pressure of water but only weakly upon the velocity of droplets through the Sherwood number.

In general, the most significant disagreement between the model simulations and experimental measurements is the vertical distance fallen by the droplet. As discussed above, the FDC is not a completely airtight system, having a small aperture at the top into which the droplets are dispensed. This is not fully accounted for in the calculation of the gas flow velocity leading to a small but constant error in the gas flow velocity input parameter to the model.

**Figure 10:**
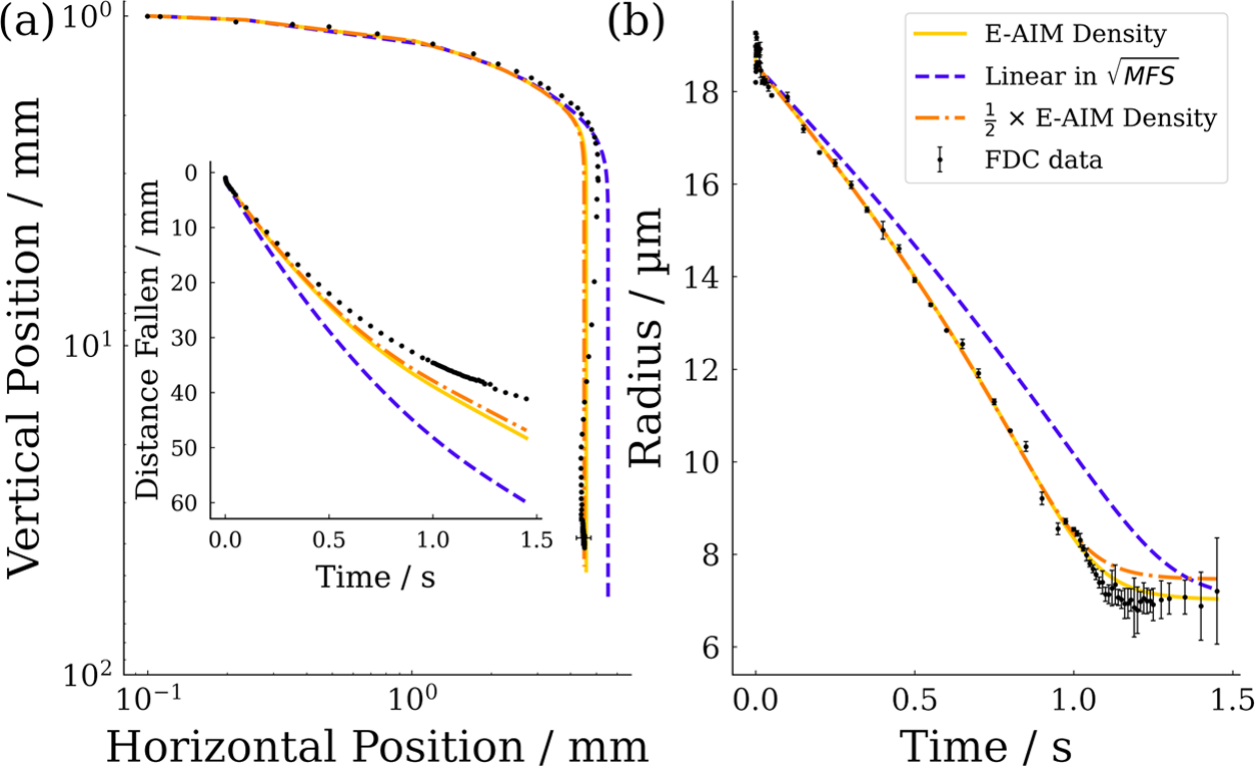
Sensitivity analysis of SADKAT simulations to input parameterization of solution density corresponding to those shown in Figure 2 with FDC data shown for comparison. (a) Spatial trajectory with inset of distance fallen over time. (b) Evaporation profile.

The model is sensitive to the parameterizations of droplet properties as well as experimental factors. Figure 10 displays the sensitivity of the model to the parameterizations of solution density shown in Figure 2a. The density of solution is shown to affect both the droplet trajectory and evaporation profile, with the simplest parameterization clearly deviating from experimental data early in the droplet lifetime. The linear parameterization of density with respect to MFS^1/2^ increases greatly at a low MFS relative to the E-AIM parameterization, which results in droplets showing a larger relaxation time, traveling further in the horizontal direction. As the density of the particle also features in the denominator of the first term in eq S2, droplet temperature and consequently evaporation rate is modified by different density parameterizations. The parameterization using half the E-AIM value is seen to affect the simulation most at the late stages of droplet drying when the predicted densities become increasingly separated. Therefore, little deviation in trajectory is observed and most of the evaporative behavior is well represented, with significant deviation only resolvable from ∼ 1 s.

**Figure 11:**
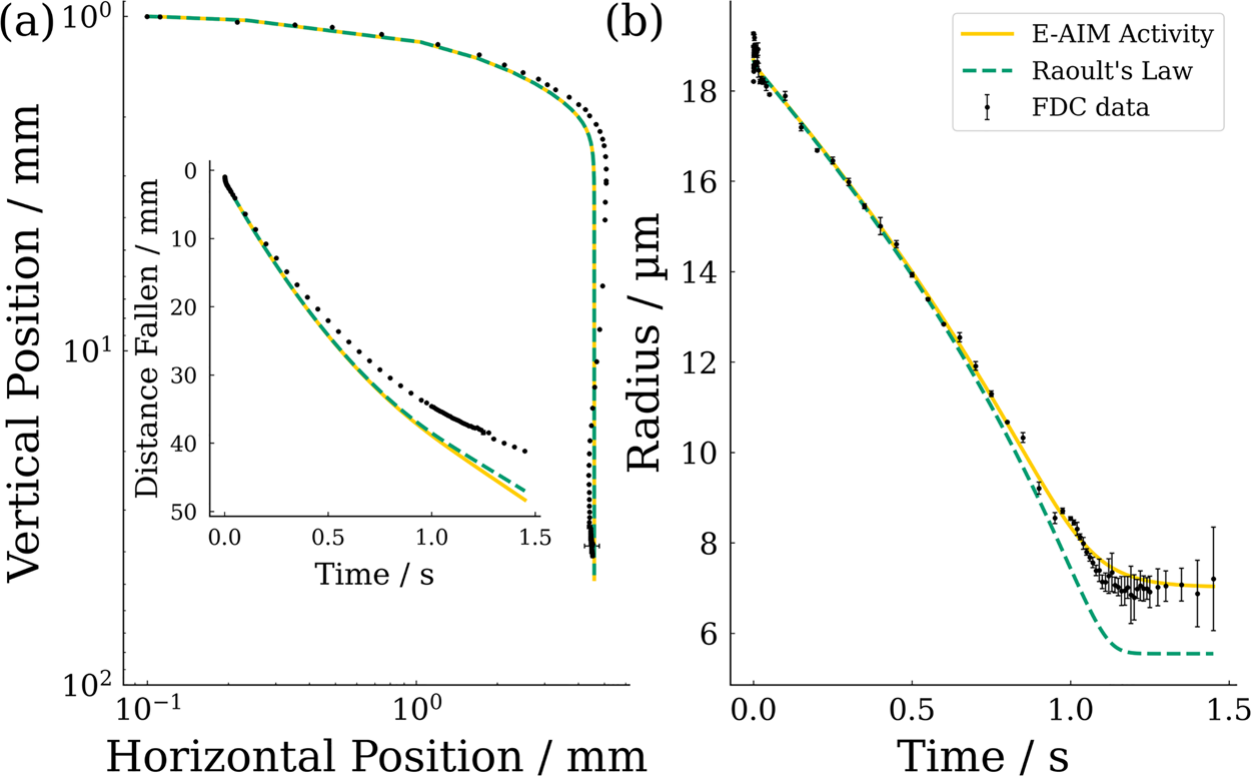
Sensitivity analysis of SADKAT simulations to input parameterization of water activity depending on solution composition corresponding to those shown in Figure 2 with FDC data shown for comparison. (a) Spatial trajectory with inset of distance fallen over time. (b) Evaporation profile.

Figure 11 displays the sensitivity of the model to the parameterization of the water activity used, either Raoult’s law or E-AIM data. Raoult’s law represents the initial droplet evaporation well, only deviating significantly from the measurement after approximately 0.7 s, when the MFS is approximately 0.18 (*a*_w_ = 0.86 according to the E-AIM-based data). In Figure 2b, the two parameterizations of a_w_ can be seen to diverge significantly above MFS values of about 0.2, explaining the increased difference between the two simulation results in Figure 11. The difference in radial evolution becomes apparent in the trajectory when considering the distance fallen over time, with the total horizontal distance traveled unaffected. The calculation of a final equilibrium size is significantly different depending upon the parameterization of water activity used, though it is worth noting this is ignoring any influence of crystallization.

### 4.4 Calculating an Evolving Péclet Number

**Figure 12:**
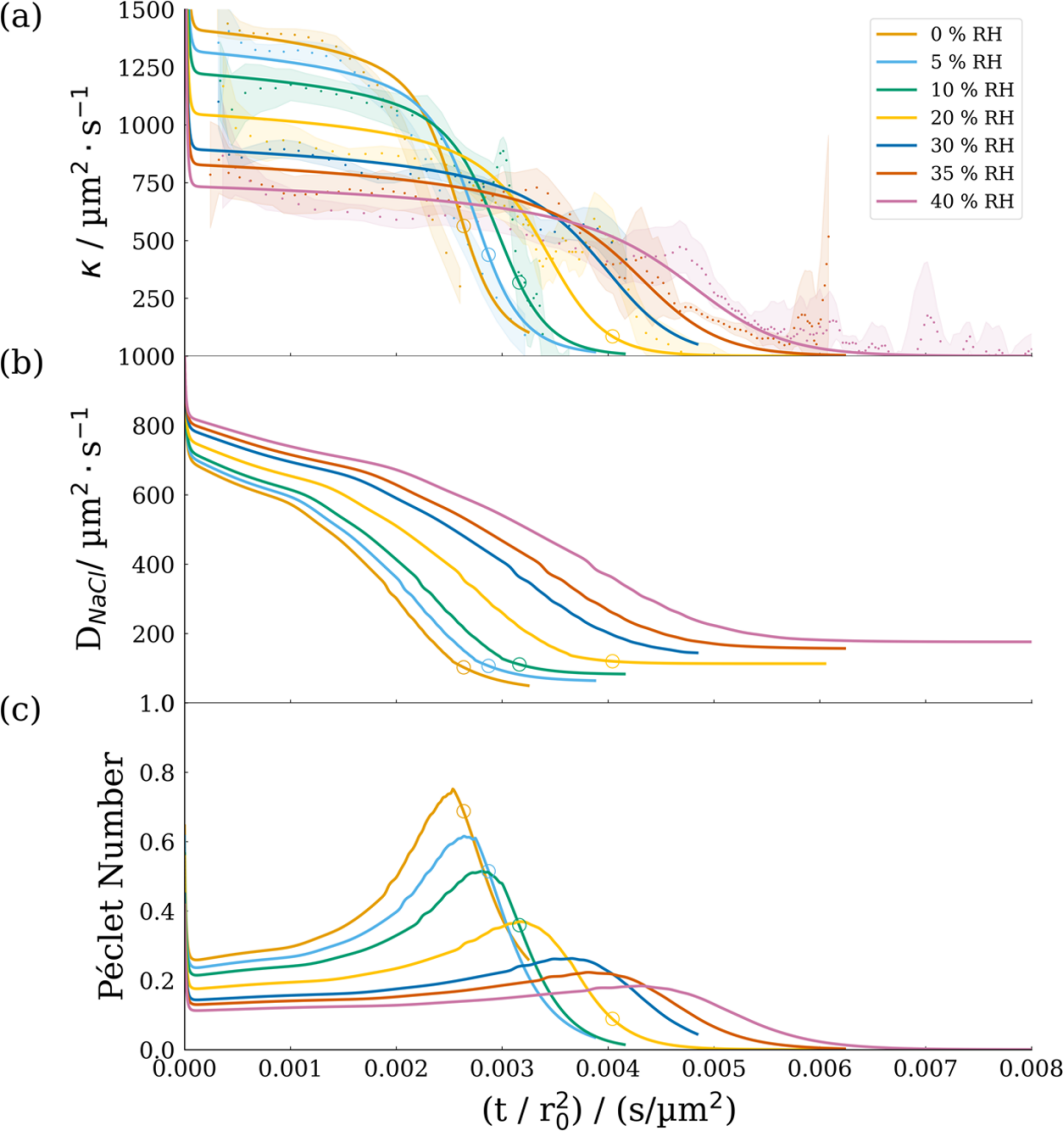
Properties of evaporating NaCl solution droplets simulated using SADKAT. (a) Evaporation rate and comparison with measured values (points with shaded error envelope). (b) Diffusion constant calculated as a function of droplet temperature and concentration. (c) Péclet number calculated using eq 4 based on the values in (a) and (b). Hollow circles indicate the point of crystallization as predicted by the model, as also indicated in Figure 7.

Unlike a pure solvent droplet, the equilibrium vapor pressure of a solution droplet changes with changing solute concentration; therefore, a time-dependent evaluation of evaporation rate must be made. Such an approach, although more complex, does allow the agreement between the simulations and experiments to be explored in more detail. The simulated and measured evolution of the evaporation rate, as calculated in eq 3, are compared in Figure 12a and have been plotted against time normalized by initial radius squared for direct comparison. Experimental and simulated evaporation rates can be seen to exhibit general agreement, both in absolute values and in temporal evolution, across a broad range of environmental conditions, despite some noise in the experimental data, associated with the variation in stroboscopic FDC measurements. As expected, dryer conditions are characterized by higher initial evaporation rates, followed by sharper reductions as the drying is completed more rapidly compared to more humid experimental conditions.

An advantage of accurate model simulations, like those presented in this work, is the ability to calculate time-dependent physical properties. For example, the evolving Péclet number, *Pé*, can be calculated over the course of the experiment from the droplet evaporation rate, κ, and the internal solute diffusion (in this case NaCl), *D*_NaCl_, both of which are functions of droplet temperature and composition, as shown in eq 4.
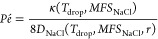
4When *D*_NaCl_ is much larger than κ, then *Pé* ≪1 and a droplet maintains compositional homogeneity. However, when the evaporation rate dominates, *Pe* ≫ 1 and surface enrichment occurs and the assumption of homogeneity in the model is no longer valid. Surface enrichment has a positive feedback effect; increases in the solute concentration and viscosity near the droplet surface lead to a further reduction in *D*_NaCl_ at the surface and further surface enrichment. The temperature dependence of *D*_NaCl_ and MFS are calculated from literature values.^19,52^Figure 12a–c shows the simulated κ, *D*_NaCl_, and *Pé* values, respectively, for droplet evaporation measurements described in Table 2.

The degree to which the model deviates from reality will be determined by the level of surface enrichment that occurs. When *Pé* ≫ 1, this is significant, but surface enrichment may even occur in systems that exhibit an initial Péclet number of less than 0.1.^19^ The Péclet number presented in Figure 12c is based on the assumption that droplets are homogeneous, which is likely not the case. As with other dimensionless numbers, the Péclet number provides insightful descriptions in extreme cases, but close to 1, the system may be considered in a transition regime. The Péclet number does however still inform understanding of final particle morphologies as the level of surface enrichment impacts the particle formation process and resulting morphology.

### 4.5 Relating Drying Rate to Final Particle Morphology

**Figure 13:**
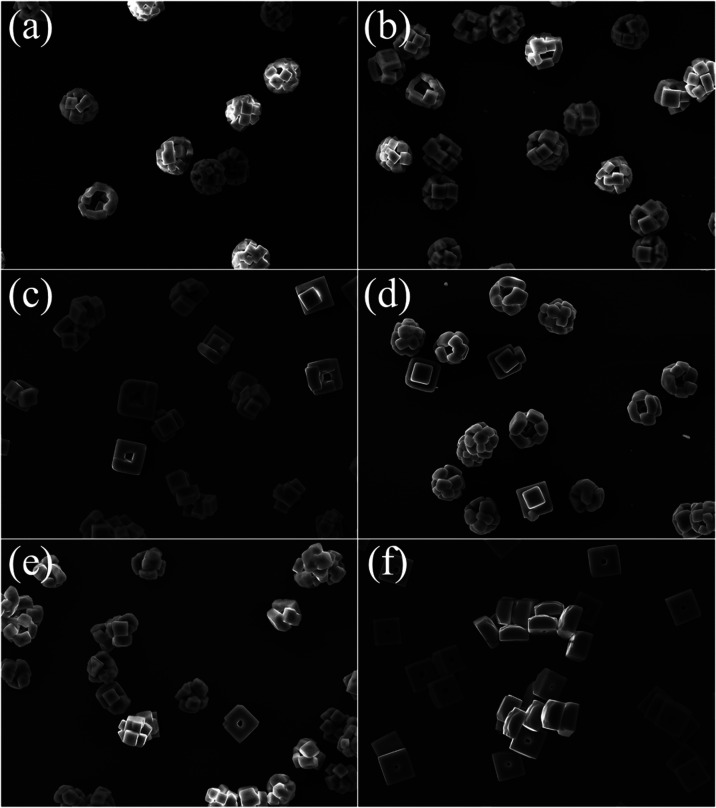
SEM images of NaCl particles formed through drying in the FDC at 294 K and varying RH values: (a) 0%, (b) 5%, (c) 10%, (d) 20%, (e) 30%, and (f) 40%.

The morphology of the final dried particles generated from the FDC can be directly related to the drying process and Péclet number. Figure 13 shows SEM images of the dry particles collected from the FDC measurements described above. The particles exhibit the same trends shown by Hardy et al., with fast-drying droplets resulting in crusts comprising higher numbers (∼10) of smaller (≤5 μm diameter) crystals and particles formed from slower drying made of a smaller number of crystals which are larger.^26^ In the case of droplets drying close to the efflorescence RH (35 and 40% RH), droplets produce a single large (∼15 μm diameter) crystal. Between these extremes in RH a more diverse range of morphologies is observed, which may be due to the stochastic nature of particle nucleation and the window of crystallization that broadens with decreased evaporation rate.^20,26^

The relationship between final morphology and drying rate can be related to the evolution of *Pé* and appears to be governed by the interplay between surface enrichment, nucleation rate, and crystal growth rates. Nucleation rates scale dramatically with solution supersaturation.^53^ Experiments at low RH values produce high evaporation rates where surface enrichment is significant and the nucleation rate will increase significantly at the surface. In such cases, the likelihood of multiple nucleation events occurring within a very short time (effectively simultaneously) is high. These crystals grow at the receding droplet surface until they make contact with each other, locking the surface structure and leaving the solvent to evaporate around them from within the particle structure. Experiments that exhibit lower evaporation rates produce less surface enrichment, meaning the surface saturation of salt is much lower with a correspondingly lower nucleation rate. With a lower nucleation rate, the probability that multiple nucleation events will occur at the same time is small, allowing the first nucleation site to grow into a crystal. As the first crystal grows, there will be a reduction in the concentration of the solution, further reducing the likelihood of other nucleation events. It is proposed that this competition could scale with evaporation rates higher than those in this work to a crust formation when very many crystals may nucleate but do not have time to gain a well-defined shape prior to the surface locking. Comparison between the dry particle morphologies shown in Figure 13 and the calculated evolution of *Pé* in Figure 12c indeed shows the expected trend. The lowest RH (highest Pé) particles exhibit multicrystal morphologies, with a structure defined by crystals of varying size arranged in a spherical shell, such as those seen in Figure 13a. Higher-RH (lower Pé) particles exhibit fewer crystals per particle, to the extreme point of a single crystal particle as shown in Figure 13f.

## 5 Conclusions

This work has presented an updated single droplet coupled evaporation/condensation and transport model and compared it against the most accurate single droplet evaporation measurements available. We have demonstrated that the model captures the evaporative behavior observed in measurements of both “stationary” droplets and falling droplets. In particular, the model accurately simulates droplet evaporation and transport mechanisms and is capable of evaluating droplet temperature suppression. The sensitivity of the model to uncertainties in input values, such as environmental RH, and important parameterization, such as water activity in solution, have been compared against experimental results. It is shown that the parameterization used and the initial values used resulted in simulations that matched measured evaporation and droplet transport well, with only vertical position over time deviating from experiments which is attributed to a systematic uncertainty in the experimental gas flow speed used.

It is noted that the predicted final size of droplets, ignoring crystallization, is similar to the measured geometric size of final crystalline particles of NaCl. As the model does not include descriptions of solidification behavior, this similarity is not considered to be due to the model being fully physically realistic.

Further work is required to simulate a wider range of systems and understand the impact of the assumed homogeneity within the model in more detail. The integration of radially resolved droplet composition models would be a significant step toward a full description of droplet behavior. Indeed, the model is not capable of representing systems with colloidal inclusions and future work will be focussed on addressing this, widening the industrial applicability of model implementations such as this.

## Supporting Information Available

Core differential equations used in the model presented, symbols and definitions and a description of a methodology for the calculation of the experimental RH from the evaporation of pure water at a given temperature, and a full set of experimental results corresponding to those shown in Figure 7 (PDF)
