# Supplementary material for: Accurate Measurements and Simulations of the Evaporation and Trajectories of Individual Solution Droplets
Source: J Phys Chem B. 2023 Apr 7;127(15):3416–30. doi: 10.1021/acs.jpcb.2c08909 (PMC10123666; doi:10.1021/acs.jpcb.2c08909)
Supplement: Supplementary file 1 — jp2c08909_si_001.pdf [file jp2c08909_si_001.pdf]

Accurate Measurements and Simulations of the Evaporation and Trajectories of Individual Solution Droplets

Daniel A. Hardy[1], Joshua F. Robinson[2,3], Thomas G. Hilditch[1], Edward Neal[1], Pascal Lemaitre[4], Jim S. Walker[1], Jonathan P. Reid[1]*

Corresponding author email: j.p.reid@bristol.ac.uk

1. School of Chemistry, University of Bristol, Bristol, BS8 1TS, United Kingdom
2. H. H. Wills Physics Laboratory, University of Bristol, Bristol BS8 1TL, United Kingdom
3. Institut für Physik, Johannes Gutenberg-Universität Mainz, Staudingerweg 7-9, 55128 Mainz, Germany
4. Institut de Radioprotection et de Sûreté Nucléaire (IRSN), PSN-RES, SCA, LPMA, Gif sur Yvette, France

Supplementary Information

Equations S1 – S4 describe the evolution of droplet mass, m_p, droplet temperature, T_p, droplet velocity, V_p, and droplet position, x_p, with respect to time, t, respectively. A full definition of the symbols used in these equation is presented in Table S1

$$\frac{dm_p}{dt} = 4\pi r_p \rho_g \left(\frac{M_{sol}}{M_g}\right) C D_\infty Sh\ ln\left(\frac{p - p_{v,a}}{p - p_{v,\infty}}\right)\beta_{FS} = f_1(r_p, T_p, V_p) \quad 2$$

$$\frac{dT_p}{dt} = 3K_g \frac{T_\infty - T_p}{c_p \rho_p r_p^2} Nu + \frac{L_v \frac{dm_p}{dt}}{c_p m_p} - \frac{3\Gamma(T_\infty - T_p)}{c_p m_p r_p} = f_2(r_p, T_p, V_p) \quad 3$$

$$\frac{dV_p}{dt} = g\left(1 - \frac{\rho_p}{\rho_g}\right) - \frac{3C_d \rho_g |V_p - V_g|(V_p - V_g)}{8\rho_p r_p} = f_3(r_p, T_p, V_p) \quad 4$$

$$\frac{dx_p}{dt} = V_p = f_4(V_p) \quad 5$$

Symbol	Description	Units
∞ (subscript)	Indicates quantity relates to gas phase	-
a (subscript)	Indicates quantity relates to gas at droplet surface	-
C	Binary diffusion coefficient temperature correction	-

C_d	Drag coefficient	-
C_p	Particle specific heat capacity	$J.kg^{-1}.K^{-1}$
D	Binary diffusion coefficient	$m^2.s^{-1}$
g	Acceleration due to gravity	$m.s^{-2}$
K_g	Gas thermal conductivity	$W.m^{-1}.K^{-1}$
Kn	Knudsen number	-
L_v	Latent heat of vapourisation	$J.K^{-1}$
M_g	Gas molar mass	$kg.mol^{-1}$
m_p	Particle mass	kg
M_{sol}	Solvent molar mass	$kg.mol^{-1}$
Nu	Nusselt number	-
p	Pressure	Pa
Pé	Péclet number	-
p_v	Solvent vapour pressure	Pa
r_p	Particle radius	m
Sh	Sherwood number	-
T	Temperature	K
t	Time	s
V_g	Gas velocity	$m.s^{-1}$
V_p	Particle velocity	$m.s^{-1}$
β_{FS}	Fuchs-Sutugin correction factor	-
Γ	Stefan Boltzmann constant	$W.m^{-2}.K^{-4}$
κ	Evaporation rate	$m^2.s^{-1}$
λ	Gas mean free path	m
ρ_g	Gas density	$kg.m^{-3}$
ρ_p	Particle density	$kg.m^{-3}$

Table S1. A full list of symbols used in Equations S1 – S4

Estimation of experimental RH from droplet evaporation rates using SADKAT

After experimental validation, it is possible to use the SADKAT model to estimate the experimental RH from the evaporation of a water droplet, in the same way that the Kulmala model may be used when evaporative cooling is small. This increases the range of conditions over which the evaporation kinetics of water droplets can be used to estimate environmental RH.

Evaporation rate as a function of T & RH follows the functional form of a general binary polynomial. A quadratic binary polynomial may be used to fit the data, with the functional form being shown in Equation SI-1. In Equation 8 κ is a function of temperature and relative humidity, κ(T, RH), by taking the inverse the experimental RH may be found as a function of experimental temperature and observed evaporation rate, RH(κ, T). The CK-EDB chamber temperature may be easily measured using a thermocouple probe.

$$\kappa = C_0 + C_1 T + C_2 RH + C_3 TRH + C_4 T^2 + C_5 RH^2 \qquad \text{S-5}$$

The use of SADKAT model data can therefore enable accurate and fast calculation of CK-EDB experimental conditions.

A full set of experimental and simulated results for the evaporation of free falling sodium chloride droplets, corresponding to those described in Table 2 is shown in Figure S1.

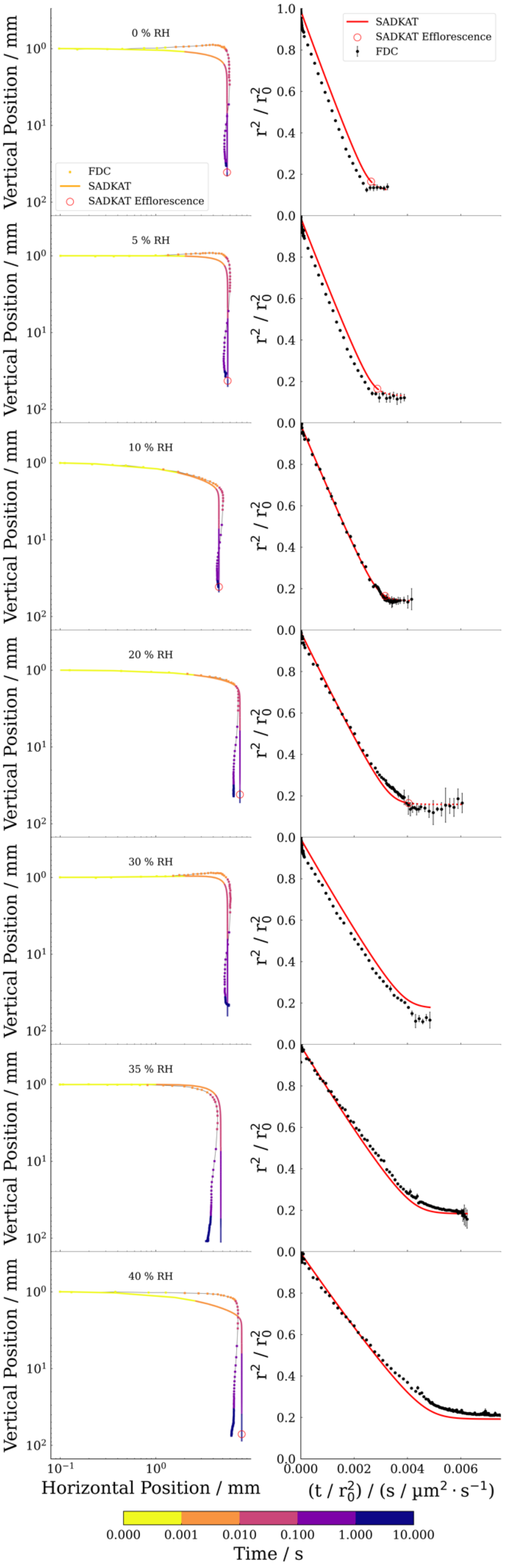

0 % RH
5 % RH
10 % RH
20 % RH
30 % RH
35 % RH
40 % RH
FDC
SADKAT
SADKAT Efflorescence
Vertical Position / mm
Horizontal Position / mm
r^2 / r_0^2
$(t / r_0^2) / (s / \mu m^2 \cdot s^{-1})$
Time / s
0.000
0.001
0.010
0.100
1.000
10.000

Figure S1. Comparative spatial trajectories (left) and evaporative profiles (right) of NaCl solution droplets (0.05 MFS) measured with the FDC and simulated using SADKAT at 294 K. The RH of each experiment is marked in the left-hand panel. The time of datapoints within the spatial trajectories are colour mapped to a logarithmic scale with transitions at 1 ms, 10 ms, 100 ms and 1 s: $t < 0.001$ s (yellow), 0.001 s < t < 0.01 s (orange), 0.01 s < t < 0.1 s (pink), 0.1 s < t < 1 s (violet), 1 s < t (indego).
